# Comparison of Bipolar Type 1 Disorder and Unipolar Mani Patients in Terms of Sociodemographic and Clinical Features and Functionality

**DOI:** 10.1192/j.eurpsy.2026.11194

**Published:** 2026-07-31

**Authors:** E. İzmir Soygenç, Ö. Şahmelikoğlu Onur

**Affiliations:** 1 Psychiatry, Bakırköy Prof Dr Mazhar Osman Research and Training Hospital, İstanbul; 2 Tekirdağ Namık Keml University, Tekirdağ, Türkiye

## Abstract

**Introduction:**

Kleist, Leonhard, and Neele differentiated bipolar and unipolar disorders, defining “pure mania” and “pure melancholia” as distinct entities. Although the concept of unipolar mania (UM) has been debated due to its rarity and unclear diagnostic criteria, studies suggest it differs from bipolar disorder (BD) in epidemiology, clinical features, and treatment response. Functionality and social adjustment tend to be better in UM. Considering these differences, further studies comparing UM, BD, and healthy controls regarding functionality and clinical characteristics are needed to clarify UM’s nosological status.

**Objectives:**

The aim of this study to determine whether there are differences between Unipolar Mania (UM), Bipolar Disorder (BD) type 1 patients in the euthymic period and healthy individuals in terms of sociodemographic characteristics, clinical features and functionality.

**Methods:**

58 patients diagnosed with euthymic BD -1, 58 patients with a diagnosis of euthymic unipolar mania, who had no depressive episode in the past, were followed up for at least 4 years, had at least 4 manic episodes, 58 healthy controls with patients for age and sex were included in the study. Sociodemographic Data Form, Young Mania Rating Scale (YMRS), Beck Depression Inventory (BDI), Positive and Negative Syndrome Scale (PANSS), Beck Anxiety Inventory (BAI), Bipolar Disorder Functioning Scale (BDFS), Functioning Assessment Short Test (FAST) were applied to the participants.

**Results:**

The presence of psychotic symptoms in the first episode were higher in the UM group than in the BD group (p<0.05). The number of total episodes were significantly higher in the BD group than in the UM group (p<0.05). The number of mania episodes, the total mean duration of episodes, the number of hospitalizations, and the use of valproate as a mood stabilizer were significantly higher in the UM group than in the BD group (p<0.05). Medical diseases both in own and family history of patients’ were higher in the BD group than in the UM group (p<0.05). Suicidality was higher in the BD group than in the UM group (p<0.05). The BD group had a higher score in the occupational functioning subscale, which is a subscale of FAST. The scores of the UM group were higher than the BD group in the emotional functioning and social withdrawal subscales, which are subscales of BDFS. (p<0.05)

**Conclusions:**

As a result of the findings of our study, the presence of differences in sociodemographic and clinical features and functionality between UM and BD supports the possibility of UM as a different diagnosis from BD.

**Disclosure of Interest:**

None Declared

